# Human-induced pluripotent stem cell-derived ovarian support cell co-culture improves oocyte maturation *in vitro* after abbreviated gonadotropin stimulation

**DOI:** 10.1093/humrep/dead205

**Published:** 2023-10-10

**Authors:** Sabrina Piechota, Maria Marchante, Alexa Giovannini, Bruna Paulsen, Kathryn S Potts, Graham Rockwell, Caroline Aschenberger, Alexander D Noblett, Alexandra B Figueroa, Marta Sanchez, Ferran Barrachina, Klaus Wiemer, Luis Guzman, Pedro Belchin, Merrick Pierson Smela, Patrick R J Fortuna, Pranam Chatterjee, Nam D Tran, Dawn A Kelk, Marcy Forti, Shelby Marcinyshyn, Trozalla Smith, David H McCulloh, Marta-Julia Fernandez-Gonzalez, Baruch Abittan, Silvia Ortiz, Joshua U Klein, Peter Klatsky, Daniel Ordonez-Perez, Christian C Kramme

**Affiliations:** Gameto Inc., New York, NY, USA; Gameto Inc., New York, NY, USA; Gameto Inc., New York, NY, USA; Gameto Inc., New York, NY, USA; Gameto Inc., New York, NY, USA; Gameto Inc., New York, NY, USA; Gameto Inc., New York, NY, USA; Gameto Inc., New York, NY, USA; Gameto Inc., New York, NY, USA; Ruber Juan Bravo University Hospital, Eugin Group, Madrid, Spain; Gameto Inc., New York, NY, USA; KEW Technology, Seattle, WA, USA; Pranor Clinic, Lima, Peru; Ruber Juan Bravo University Hospital, Eugin Group, Madrid, Spain; Wyss Institute, Harvard Medical School, Boston, MA, USA; Department of Genetics, Harvard Medical School, Boston, MA, USA; Wyss Institute, Harvard Medical School, Boston, MA, USA; Department of Genetics, Harvard Medical School, Boston, MA, USA; Department of Biomedical Engineering, Duke University, Durham, NC, USA; Department of Computer Science, Duke University, Durham, NC, USA; Spring Fertility, New York, NY, USA; Extend Fertility, New York, NY, USA; Extend Fertility, New York, NY, USA; Extend Fertility, New York, NY, USA; Spring Fertility, New York, NY, USA; Gameto Inc., New York, NY, USA; Biogenetics Corporation, Mountainside, NJ, USA; Sperm and Embryo Bank of New York, New York, NY, USA; Biogenetics Laboratory, Brooklyn, NY, USA; ReproART, Georgian American Center for Reproductive Medicine, Tbilisi, GA, USA; Gameto Inc., New York, NY, USA; Extend Fertility, New York, NY, USA; Pranor Clinic, Lima, Peru; Extend Fertility, New York, NY, USA; Spring Fertility, New York, NY, USA; Ruber Juan Bravo University Hospital, Eugin Group, Madrid, Spain; Gameto Inc., New York, NY, USA

**Keywords:** ovarian support cells, *in vitro* maturation, stem cells, blastocysts, abbreviated stimulation, oocyte quality, granulosa cells, embryos, euploidy

## Abstract

**STUDY QUESTION:**

Can *in vitro* maturation (IVM) and developmental competence of human oocytes be improved by co-culture with ovarian support cells (OSCs) derived from human-induced pluripotent stem cells (hiPSCs)?

**SUMMARY ANSWER:**

OSC-IVM significantly improves the rates of metaphase II (MII) formation and euploid Day 5 or 6 blastocyst formation, when compared to a commercially available IVM system.

**WHAT IS KNOWN ALREADY:**

IVM has historically shown highly variable performance in maturing oocytes and generating oocytes with strong developmental capacity, while limited studies have shown a positive benefit of primary granulosa cell co-culture for IVM. We recently reported the development of OSCs generated from hiPSCs that recapitulate dynamic ovarian function *in vitro*.

**STUDY DESIGN, SIZE, DURATION:**

The study was designed as a basic science study, using randomized sibling oocyte specimen allocation. Using pilot study data, a prospective sample size of 20 donors or at least 65 oocytes per condition were used for subsequent experiments. A total of 67 oocyte donors were recruited to undergo abbreviated gonadotropin stimulation with or without hCG triggers and retrieved cumulus–oocyte complexes (COCs) were allocated between the OSC-IVM or control conditions (fetal-like OSC (FOSC)-IVM or media-only IVM) in three independent experimental design formats. The total study duration was 1 April 2022 to 1 July 2023.

**PARTICIPANTS/MATERIALS, SETTING, METHODS:**

Oocyte donors between the ages of 19 and 37 years were recruited for retrieval after informed consent, with assessment of anti-Mullerian hormone, antral follicle count, age, BMI and ovarian pathology used for inclusion and exclusion criteria. In experiment 1, 27 oocyte donors were recruited, in experiment 2, 23 oocyte donors were recruited, and in experiment 3, 17 oocyte donors and 3 sperm donors were recruited. The OSC-IVM culture condition was composed of 100 000 OSCs in suspension culture with hCG, recombinant FSH, androstenedione, and doxycycline supplementation. IVM controls lacked OSCs and contained either the same supplementation, FSH and hCG only (a commercial IVM control), or FOSCs with the same supplementation (Media control). Experiment 1 compared OSC-IVM, FOSC-IVM, and a Media control, while experiments 2 and 3 compared OSC-IVM and a commercial IVM control. Primary endpoints in the first two experiments were the MII formation (i.e. maturation) rate and morphological quality assessment. In the third experiment, the fertilization and embryo formation rates were assessed with genetic testing for aneuploidy and epigenetic quality in blastocysts.

**MAIN RESULTS AND THE ROLE OF CHANCE:**

We observed a statistically significant improvement (∼1.5×) in maturation outcomes for oocytes that underwent IVM with OSCs compared to control Media-IVM and FOSC-IVM in experiment 1. More specifically, the OSC-IVM group yielded a MII formation rate of 68% ± 6.83% SEM versus 46% ± 8.51% SEM in the Media control (*P *=* *0.02592, unpaired *t-*test). FOSC-IVM yielded a 51% ± 9.23% SEM MII formation rate which did not significantly differ from the media control (*P *=* *0.77 unpaired *t*-test). Additionally, OSC-IVM yielded a statistically significant ∼1.6× higher average MII formation rate at 68% ± 6.74% when compared to 43% ± 7.90% in the commercially available IVM control condition (*P *=* *0.0349, paired *t-*test) in experiment 2. Oocyte morphological quality between OSC-IVM and the controls did not significantly differ. In experiment 3, OSC-IVM oocytes demonstrated a statistically significant improvement in Day 5 or 6 euploid blastocyst formation per COC compared to the commercial IVM control (25% ± 7.47% vs 11% ± 3.82%, *P *=* *0.0349 logistic regression). Also in experiment 3, the OSC-treated oocytes generated blastocysts with similar global and germline differentially methylated region epigenetic profiles compared commercial IVM controls or blastocysts after either conventional ovarian stimulation.

**LARGE SCALE DATA:**

N/A.

**LIMITATIONS, REASONS FOR CAUTION:**

While the findings of this study are compelling, the cohort size remains limited and was powered on preliminary pilot studies, and the basic research nature of the study limits generalizability compared to randomized control trials. Additionally, use of hCG-triggered cycles results in a heterogenous oocyte cohort, and potential differences in the underlying maturation state of oocytes pre-IVM may limit or bias findings. Further research is needed to clarify and characterize the precise mechanism of action of the OSC-IVM system. Further research is also needed to establish whether these embryos are capable of implantation and further development, a key indication of their clinical utility.

**WIDER IMPLICATIONS OF THE FINDINGS:**

Together, these findings demonstrate a novel approach to IVM with broad applicability to modern ART practice. The controls used in this study are in line with and have produced similar to findings to those in the literature, and the outcome of this study supports findings from previous co-culture studies that found benefits of primary granulosa cells on IVM outcomes. The OSC-IVM system shows promise as a highly flexible IVM approach that can complement a broad range of stimulation styles and patient populations. Particularly for patients who cannot or prefer not to undergo conventional gonadotropin stimulation, OSC-IVM may present a viable path for obtaining developmentally competent, mature oocytes.

**STUDY FUNDING/COMPETING INTEREST(s):**

A.D.N., A.B.F., A.G., B.P., C.A., C.C.K., F.B., G.R., K.S.P., K.W., M.M., P.C., S.P., and M.-J.F.-G. are shareholders in the for-profit biotechnology company Gameto Inc. P.R.J.F. declares paid consultancy for Gameto Inc. P.C. also declares paid consultancy for the Scientific Advisory Board for Gameto Inc. D.H.M. has received consulting services from Granata Bio, Sanford Fertility and Reproductive Medicine, Gameto, and Buffalo IVF, and travel support from the Upper Egypt Assisted Reproduction Society. C.C.K., S.P., M.M., A.G., B.P., K.S.P., G.R., and A.D.N. are listed on a patent covering the use of OSCs for IVM: U.S. Provisional Patent Application No. 63/492,210. Additionally, C.C.K. and K.W. are listed on three patents covering the use of OSCs for IVM: U.S. Patent Application No. 17/846,725, U.S Patent Application No. 17/846,845, and International Patent Application No.: PCT/US2023/026012. C.C.K., M.P.S., and P.C. additionally are listed on three patents for the transcription factor-directed production of granulosa-like cells from stem cells: International Patent Application No.: PCT/US2023/065140, U.S. Provisional Application No. 63/326,640, and U.S. Provisional Application No. 63/444,108. The remaining authors have no conflicts of interest to declare.

## Introduction

It is estimated that nearly 1 in 6 women in the USA and in many European nations have sought fertility treatment (Osterman *et al.*, 2023). ARTs such as IVF offer remarkable benefits for treating aspects of infertility for certain patient populations (Fauser, 2019; Gaskins *et al.*, 2023). However, the use of high doses of gonadotropin in stimulation regimens has resulted in uncomfortable and sometimes serious and long lasting side effects for women undergoing the process, with rising costs continuing to limit access in many nations (El Tokhy *et al.*, 2016; Ethics Committee of the American Society for Reproductive Medicine, 2021). In addition, for some women for whom conventional ovarian stimulation (COS) is contraindicated, standard IVF practice is not feasible due to the medical risk it poses (De Vos *et al.*, 2014, 2021; Braam *et al.*, 2020). Therefore, new technologies that allow for a reduction of gonadotropin stimulation in fertility treatments are important to improve patient outcomes and ART accessibility.

One option for reducing gonadotropin usage in fertility treatment is through the application of minimal or abbreviated stimulation cycles. Such cycles drastically reduce the cost and complications of IVF, while nearly completely eliminating the chance for severe complications such as ovarian hyperstimulation syndrome (Walls *et al.*, 2015; Zhang *et al.*, 2016; Vuong *et al.*, 2020a). Depending on the protocol, abbreviated gonadotropin stimulation results in a cohort of oocytes that are mostly or all immature, including germinal vesicle (GV) containing oocytes and oocytes lacking a GV and a polar body (PB) (metaphase I, MI), while some regimens may yield a small pool of mature oocytes with a PB (metaphase II, MII) (Son and Tan, 2010; Sánchez *et al.*, 2017; Vuong *et al.*, 2020a). In abbreviated gonadotropin cycles, *in vitro* maturation (IVM) holds promise as an ART strategy to mature oocytes outside of the body for use in fertility treatments. Numerous studies have employed IVM effectively, often through the application of reproductive cell culture media supplemented with growth factors, small molecules, and gonadotropins (Shu-Chi *et al.*, 2006; Fadini *et al.*, 2009; De Vos *et al.*, 2011; Moschini *et al.*, 2011; Guzman *et al.*, 2012; Pongsuthirak *et al.*, 2015; Sánchez *et al.*, 2017; Braam *et al.*, 2019; Sanchez *et al.*, 2019; Ma *et al.*, 2020; Vuong *et al.*, 2020a,b; Akin *et al.*, 2021; Grynberg *et al.*, 2022; Mohsenzadeh *et al.*, 2022). However, many of these studies have shown mixed results of IVM applications in terms of the rates of oocyte maturation, cleaved embryo formation, and blastocyst formation, with some studies showing lower pregnancy rates from IVM cycles. Therefore, improvement is needed in IVM strategies that yield robust, high-quality oocytes with strong developmental competence.

Oocyte maturation is a coordinated series of molecular processes occurring within the oocyte nucleus and cytoplasm (Edwards, 1965; Strączyńska *et al.*, 2022). These molecular processes result in both nuclear maturation, namely extrusion of the first PB (PB1), assembly of the second meiotic spindle and cytoplasmic maturation, defined as distribution of organelles in the oocyte cytoplasm and deposition of proteins and transcripts needed for fertilization competence and subsequent embryogenesis (The Ovary, 2019). Oocyte maturation normally occurs within the ovary in response to complex, temporally-regulated, extrinsic signals provided through hormone signaling, growth factor production, and nutrient dynamics in the follicular environment (Erickson and Shimasaki, 2001; Nilsson and Skinner, 2001; Park *et al.*, 2004; Hsieh *et al.*, 2009; Liu *et al.*, 2017; Fontana *et al.*, 2020; Zhang *et al.*, 2020; Strączyńska *et al.*, 2022). Many of these processes are regulated by somatic ovarian cells, such as granulosa, theca, and stroma cells. Limited but promising research has been performed to assess the use of these somatic cell types to support human oocyte IVM applications (Torre *et al.*, 2006; Johnson *et al.*, 2008; Jahromi *et al.*, 2015; Zgórecka *et al.*, 2022). However, the use of patient-derived somatic cell types besides cumulus cells is challenging due to inconsistent cell type composition retrievals, difficulties in extracting and purifying cell populations, and the infeasibility of extracting sufficient support cells without harming patients. In addition, for some patients, their infertility may arise from inappropriate signaling within and among their own follicular cells, limiting their utility for IVM co-culture. Therefore, new IVM systems that effectively and consistently deliver specific ovarian support cell (OSC) types for co-culture with immature oocytes is a salient potential platform for human oocyte IVM.

Our previous work described the generation of OSCs from human-induced pluripotent stem cells (hiPSCs) in a rapid, efficient, and reproducible manner through transcription factor (TF)-directed differentiation (Pierson Smela *et al.*, 2023). The OSCs primarily are composed of FOXL2+ AMHR2+ NR2F2+ granulosa-like cells. The OSCs are steroidogenic, producing aromatase in response to FSH stimulation to catalyze the estradiol synthesis pathway, and produce the necessary growth factors needed for robust paracrine interaction with oocytes and cumulus cells. In this study, we investigated the potential of hiPSC-derived OSCs to improve IVM of oocytes in a co-culture system with immature cumulus–oocyte complexes (COCs) retrieved from subjects following abbreviated gonadotropin cycles.

## Materials and methods

### Type of study

The study described here is a basic science study, designed to evaluate the oocyte maturation-stimulating potential of a novel IVM system and its effect on oocyte quality. No gametes or embryos utilized or created in this study were used for clinical trials or reproductive purposes such as long-term banking, transfer, implantation or gamete donation.

### Subject ages, ethics, and informed consent

This study was performed according to the ethical principles of the Declaration of Helsinki. Oocyte donor subjects were enrolled in the study through the Ruber Clinic (Madrid, Spain), Spring Fertility Clinic (New York, USA), Extend Fertility Clinic (New York, USA), and Pranor Clinic (Lima, Peru), using informed consent for donation of gametes for research purposes, with ethical approval from CNRHA 47/428973.9/22 (Spain), Western IRB No. 20225832 (USA), and Protocol No. GC-MSP-01 (Peru), respectively. Oocytes retrieved and donated from the Ruber and Pranor clinics, after consent, were utilized for research purpose for maturation analysis endpoints only, while oocytes retrieved and donated from Spring Fertility and Extend Fertility after consent, were utilized for oocyte maturation and embryo formation endpoints. Sperm donor subjects were enrolled through Seattle Sperm Bank (Seattle, Washington) after informed consent through Western IRB No. 20225832 (USA), for donation of gametes for research purposes involving the creation of human embryos. An additional eight euploid blastocysts were donated for research purposes for this study to serve as ovarian stimulation (COS) controls for embryo epigenetic analysis under the IRB No. 2022-001 (ReproART, Georgia). Subject ages ranged between 19 and 37 years of age, with an average age of 28 years.

### Experimental design

**Figure 1.**
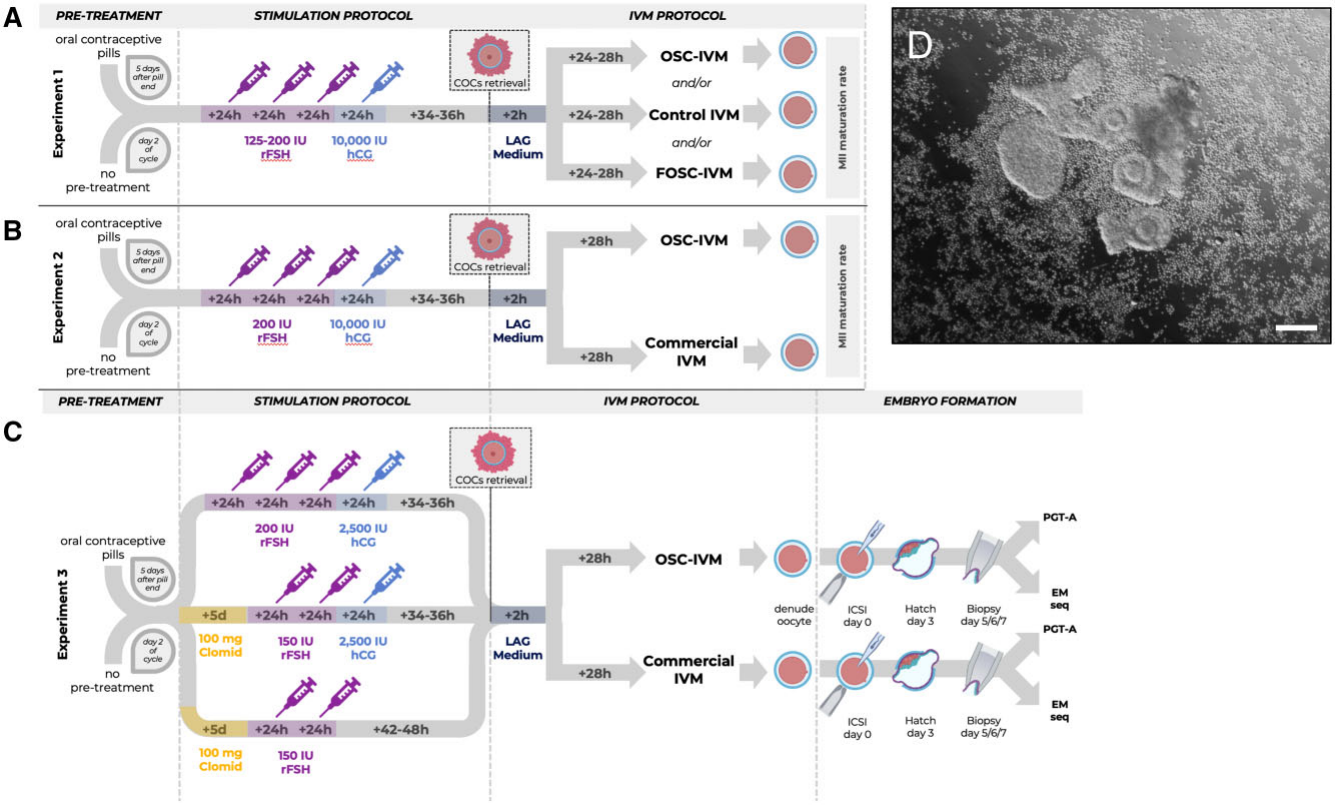
**Study design flow chart.** Schematic representation of the three independent experiments performed in the study, depicting stimulation protocols and embryology workflows. (**A**) Experiment 1 refers to Fig. 2. (**B**) Experiment 2 refers to Fig. 3. (**C**) Experiment 3 refers to Figs 4 and 5. (**D**) Representative image of co-culture setup at time of plating containing human cumulus–oocyte complexes (COCs) (n = 5) and 100 000 ovarian support cells (OSCs). Scale bar: 100 µm. COCs with varying degrees of cumulus enclosure are seen with surrounding OSCs in suspension culture. COCs, cumulus–oocytes complexes; ICSI, intracytoplasmic sperm injection; IU, international units; rFSH, recombinant follicle stimulating hormone; Clomid, clomiphene citrate; hCG, human chorionic gonadotropin; IVM, *in vitro* maturation; LAG, pre-incubation medium (MediCult); PGT-A, pre-implantation genetic testing for aneuploidy; EM-Seq; enzymatic methylation sequencing.

This study utilized three independent experiments, which tested hypotheses on non-overlapping patient groups at different clinics under different ethical approvals and stimulation regimens. The design of these experiments is shown in a flowchart in Fig. 1.

#### Experiment 1 (OSC activity)

The purpose of this comparison was to determine whether the stimulated OSCs were the active ingredient of the co-culture system in a pilot study. This experiment ended with oocyte maturation analysis and involved subjects from the Ruber and Pranor Clinic.

#### Experiment 2 (OSC clinical relevance)

The purpose of this experiment was to compare the efficacy of the OSC-IVM system and the commercially available IVM system (Medicult IVM) in maturing human oocytes. This experiment ended with oocyte maturation analysis and involved subjects from the Ruber Clinic.

#### Experiment 3 (OSC clinical relevance)

The purpose of this experiment was to compare the efficacy of the OSC-IVM system and the commercially available IVM system (Medicult IVM) in maturing human oocytes and generating euploid blastocysts. This experiment ended with embryo formation and embryo molecular analysis and involved subjects from the Spring Fertility Clinic and Extend Fertility Clinic.

### Study duration

The period of recruitment and duration of Experiment 1 were 1 April 2022 to 1 August 2022. The period of recruitment and duration of Experiment 2 were 1 October 2022 to 1 December 2022. The period of recruitment and duration of Experiment 3 were 1 December 2022 to 1 July 2023.

### Subject screening and stimulation characteristics

#### Experiment 1

A cohort of 27 subjects received 3–4 days of stimulation using 325–600 IU of recombinant FSH (rFSH) with a 10 000-IU human chorionic gonadotropin (hCG) trigger in preparation for immature oocyte aspiration. Non-contraceptive subjects began stimulation on Day 2 of their menstrual cycle, while contraceptive patients began on Day 5 following cessation of oral contraceptive pills (OCPs). Ovulation triggers were scheduled when two or more follicles reached 7–9 mm in diameter, and average follicle sizes of 6–14 mm were used for extraction 34–36 h after trigger. Inclusion criteria included: anti-Mullerian hormone (AMH) ≥1 ng/ml, antral follicle count (AFC) ≥15, ages 19–40 years, BMI 20–30 kg/m^2^, and normal ovulatory cycle. Exclusion criteria included: diagnosis of severe endometriosis, pathology preventing access to both ovaries, medical contraindication for undergoing stimulation or anesthesia, positive test for sexually transmitted disease, clinical diagnosis of polycystic ovarian syndrome (PCOS), clinical diagnosis of hypothyroidism or previously known defect in oocyte maturation.

#### Experiment 2

A cohort of 23 subjects received three consecutive days of 200 IU of rFSH with a 10 000-IU hCG trigger in preparation for immature oocyte aspiration. Non-contraceptive subjects began stimulation on Day 2 of their menstrual cycle, while contraceptive patients began on Day 5 following cessation of pills. Ovulation triggers were scheduled when two or more follicles reached 7–9 mm in diameter, and average follicle sizes of 6–14 mm were used for extraction 34–36 h after trigger. Inclusion criteria included: AMH ≥1.5 ng/ml, AFC≥15, ages 25–35 years, BMI 20–30 kg/m^2^, and normal ovulatory cycle. Exclusion criteria included: diagnosis of severe endometriosis, pathology preventing access to both ovaries, medical contraindication for undergoing stimulation or anesthesia, positive test for sexually transmitted disease, clinical diagnosis of PCOS, clinical diagnosis of hypothyroidism or previously known defect in oocyte maturation.

#### Experiment 3

A cohort of 17 subjects received either five doses of clomiphene citrate (Clomid) (100 mg) with an additional one to two doses of 150 IU rFSH with or without a 2500-IU hCG trigger or three doses of 200 IU rFSH with a 2500-IU hCG trigger. Non-contraceptive subjects began stimulation on Day 2 of their menstrual cycle, while contraceptive patients began on Day 5 following cessation of pills. Ovulation triggers were scheduled when two or more follicles reached 7–9 mm in diameter, and average follicle sizes of 6–14 mm were used for extraction 34–36 h after trigger. In non-triggered cycles, extraction was scheduled when two or more follicles reached 7–9 mm in diameter, and average follicle sizes of 6–14 mm were used for extraction 42–48 h after the last rFSH injection. Inclusion criteria included: AMH≥2 ng/ml, AFC≥20, ages 25–35 years, BMI 20–30 kg/m^2^, normal ovulatory cycle, and TSH≤3 mIU/ml. Exclusion criteria included: diagnosis of severe endometriosis, pathology preventing access to both ovaries, medical contraindication for undergoing stimulation or anesthesia, positive test for sexually transmitted disease, clinical diagnosis of PCOS, clinical diagnosis of hypothyroidism, or previously known defect in oocyte maturation. For male gamete donors, three donors aged 21–35 years, who were consented for research donation resulting in embryo formation, were selection for semen donation with the following characteristics: normal volume (>2.0 ml), normal sperm count (concentration; >15 m/ml), normal motility (>40%), and normal morphology (>4% strict morphology).

A complete table of donor stimulation regimens for each donor in the study is provided in Supplementary Table S1.

### Serum hormone and follicle development monitoring during stimulation

Baseline serum evaluation of AMH levels was performed for all patients as part of inclusion criteria screening. For Experiments 1 and 2, patients were additionally monitored on the day of stimulation start, day of trigger, and day of oocyte retrieval. During these monitoring periods, transvaginal ultrasound was performed to assess follicle sizes and development. Additionally, serum levels of LH, E2, and P4 were measured to assess the early follicular growth phase (E2 > 100 ng/ml) and to monitor for premature luteinization and for spikes in LH (LH≥10 IU/ml). For Experiment 3, which was performed at a different center to Experiments 1 and 2, ultrasound monitoring and serum evaluation of E2, P4, and LH was performed at the start of stimulation and on the day of oocyte retrieval only.

### Aspiration of small ovarian follicles to retrieve immature cumulus oocyte complexes

#### Experiments 1 and 2

Aspirations were performed 34–36 h after the trigger injection (10 000 IU hCG) using a transvaginal ultrasound with a needle guide on the probe to retrieve oocytes for co-culture experiments. Aspiration was performed using ASP medium (Vitrolife) without follicular flushing using double lumen 19-gauge needles (double lumen needles were selected due to the additional stiffness provided by the second channel inside the needle). Vacuum pump suction (100–120 mmHg) was used to harvest follicular contents through the aspiration needle and tubing into a 15-ml round bottom polystyrene centrifuge tube.

#### Experiment 3

For the conditions where the final outcome was embryo formation, aspirations were performed 34–36 h after trigger injection (2500 IU hCG) or 42–48 h after the last rFSH injection for untriggered cycles. Aspiration was performed without follicular flushing using a single lumen 17-, 19-, or 20-gauge needle with vacuum pump suction (100–120 mmHg) to harvest follicular contents into a 15-ml round bottom polystyrene centrifuge tube.

In all cases, rapid rotation of the aspiration needle around its long axis when the follicle had collapsed provided a curettage effect to assist the release of COCs. Although follicles were not flushed, the aspiration needle was removed from the subject and flushed frequently throughout the oocyte retrieval procedure to limit clotting and needle blockages.

Follicular aspirates were examined in the laboratory using a dissecting microscope. Aspirates tended to include more blood than typical IVF follicle aspirations, so samples and needles were washed with HEPES media (G-MOPS Plus, Vitrolife) to minimize clotting. Often, the aspirate was additionally filtered using a 70-micron cell strainer (Falcon, Corning) to improve the oocyte search process yield and speed. COCs were transferred using a sterile Pasteur pipette to a dish containing a pre-incubation LAG Medium (Medicult, Cooper Surgical) until use in the IVM procedure. The number of COCs aspirated was equal to roughly 40–50% of the subject’s AFC.

### Preparation of ovarian support cells (OSCs)

OSCs were created from hiPSCs according to a 5-day TF-directed protocol described previously (Pierson Smela *et al.*, 2023). The cell lines utilized in this study came from a single original cell source who consented for research use of the cells and were not generated on a per subject basis. The OSCs were produced in multiple batches and cryopreserved in vials of 120 000–150 000 live cells and stored in liquid nitrogen in CryoStor CS10 Cell Freezing Medium (StemCell Technologies). For the purposes of this study, the OSCs utilized in the OSC-IVM condition were generated through use of the TFs *NR5A1*, *RUNX2*, and *GATA4*. In Experiment 1, an additional control cell line of fetal-like ovarian somatic cells (FOSC-IVM) was generated using overexpression of the TFs *NR5A1*, *RUNX1*, *GATA4*, and *FOXL2* through methods described previously (Pierson Smela *et al.*, 2023). All OSC lines were negative for mycoplasma and human-infectious pathogens, had a freeze-thaw viability greater than 80%, were confirmed for cell identity via flow cytometry, immunofluorescence and/or qPCR of FOXL2, CD82, and NR2F2, and were validated as steroidogenic via estradiol (E2) and progesterone (P4) ELISA assays (R&D Systems).

Culture dishes (4 + 8 Well Dishes, BIRR) for oocyte maturation experiments were prepared with culture media and additional constituents in 100 µl droplets under mineral oil the day before oocyte collection, and equilibrated in the incubator overnight. On the morning of oocyte collection, cryopreserved OSCs were thawed for 2–3 min at 37 °C (in a heated bead or water bath), resuspended in OSC-IVM medium, and washed twice using centrifugation pelleting at 300×*g* for 5 min to remove residual cryoprotectant. Equilibrated OSC-IVM media was used for final cell resuspension. OSCs were then plated at a concentration of 100 000 cells per 100 µl droplet by replacing 50 µl of the droplet with 50 µl of the OSC suspension 2–4 h before the addition of oocytes to allow for culture equilibration and media conditioning (Supplementary Fig. S1). All FOSCs utilized in Experiment 1 were prepared in the same manner as OSCs.

### In vitro maturation

COCs were maintained in preincubation LAG Medium (Medicult, Cooper Surgical) at 37 °C for 2–3 h after retrieval prior to introduction to IVM conditions.

Three experimental comparisons were performed to address the following goals.

#### Experiment 1 (OSC activity)

The purpose of this comparison was to determine whether the stimulated OSCs were the active ingredient of the co-culture system versus other medium components alone or other cell types. Media were prepared following the manufacturer’s recommendations (Medicult, Cooper Surgical), and further supplemented with androstenedione and doxycycline (both necessary for activation of OSCs) in order to compare oocyte maturation outcomes with or without OSCs in the same medium formulation (Table 1).

**Table 1.** Cell culture media conditions.

	Experiment 1: OSC activity		Experiment 2: OSC clinical relevance		Experiment 3: OSC clinical relevance	
			IVM only		Embryo formation	
	Experimental groups	Control group	Experimental group	Control group	Experimental group	Control group
IVM medium (Medicult, Cooper Surgical)	X	X	X	X	X	X
10 mg/ml human serum albumin (HSA) (Life Global, GHSA-125)	X	X	X	X	X	X
75 mIU/ml of recombinant FSH (Millipore, F4021)	X	X	X	X	X	X
100 mIU/ml of recombinant hCG (Sigma, CG10)	X	X	X	X	X	X
500 ng/ml Androstenedione (Sigma, A-075l)	X	X	X		X	
1.0 µg/ml Doxycycline (StemCell Tech., 100-1047)	X	X	X		X	
100 000 cells per 100 µl droplet	X		X		X	

FSH, follicle stimulating hormone; hCG, human chorionic gonadotropin; IVM, *in vitro* maturation; OSCs, ovarian support cells.

#### Experiment 2 (OSC clinical relevance, IVM only)

The purpose of this experiment was to compare the efficacy of the OSC-IVM system and the commercially available IVM system (Medicult IVM). The Control condition was prepared and supplemented by following the manufacturer’s recommendations (Medicult, Cooper Surgical), while the medium for OSC-IVM was prepared with all supplements (Table 1).

#### Experiment 3 (OSC clinical relevance, embryo formation)

The purpose of this experiment was to compare the efficacy of the OSC-IVM system and the commercially available IVM system (Medicult IVM) in maturing human oocytes and generating euploid blastocysts. The Control condition was prepared and supplemented by following the manufacturer’s recommendations (Medicult IVM, Cooper Surgical), while medium for OSC-IVM was prepared with all supplements (Table 1). Mature oocytes from both groups were then treated identically for the subsequent embryo formation steps.

### Subject description

#### Experiment 1

We collected 179 oocytes from 27 subjects who underwent abbreviated gonadotropin stimulation, with 49 utilized in OSC-IVM co-culture, 47 utilized in FOSC-IVM co-culture, and 83 utilized in control culture. Co-culture in the experimental and control conditions was performed in parallel when possible leading to excess control samples compared to individual experimental conditions, and COCs were distributed equitably when performed in parallel, resulting in 41 unique oocyte culture setups. Equitable distribution means that COCs with distinctly large cumulus masses, small cumulus masses, or expanded cumulus masses were distributed equally between the conditions. The COCs were distributed randomly between one or two conditions, as the low number of oocytes retrieved per subject was too few to distribute oocytes between all three conditions. COCs were subjected to IVM at 37 °C for 24–28 h in a tri-gas incubator with CO_2_ adjusted so that the pH of the bicarbonate-buffered medium was 7.2–7.3, with the O_2_ level maintained at 5%.

#### Experiment 2

There were 23 subjects recruited, yielding 143 COCs with 70 utilized in the commercial IVM control and 73 utilized in the OSC-IVM condition, while two subjects were excluded from analysis due to low (<2) or no oocytes retrieved, preventing pairwise assessment. Co-culture in the experimental and control conditions was performed in parallel for all subjects. COCs were distributed equitably between the two conditions, as described above. COCs were subjected to IVM at 37 °C for 28 h in a tri-gas incubator with CO_2_ adjusted so that the pH of the bicarbonate-buffered medium was 7.2–7.3, with the O_2_ level maintained at 5%.

#### Experiment 3

IVM with subsequent embryo formation was performed to assess developmental competence of the oocytes treated in the OSC co-culture system in comparison to oocytes treated with commercially available IVM medium. There were 17 oocyte donors and three sperm donors recruited for IVM with subsequent ICSI and blastocyst formation, while three donors were excluded from analysis due to insufficient oocytes retrieved during stimulation (≤2 oocytes). There were 131 COCs included in the comparison, with 64 utilized in the commercial IVM control and 67 utilized in the OSC-IVM condition. Co-culture in the experimental and control conditions was performed in parallel. COCs were distributed equitably, as described above. COCs were subjected to IVM at 37 °C for 28 h in a tri-gas incubator with CO_2_ adjusted so that the pH of the bicarbonate-buffered medium was 7.2–7.3, with the O_2_ level maintained at 5%. Embryo formation proceeded in parallel, with the culture proceeding no longer than Day 7 post-IVM.

### Assessment of oocyte in vitro maturation and morphology

Following the 24- to 28-h IVM culture, COCs were stripped of surrounding cumulus and corona cells via hyaluronidase treatment, then oocytes were assessed for maturation state according to the following criteria: GV—presence of a GV, typically containing a single nucleolus within the oocyte; MI—absence of a GV within the oocyte and absence of a PB in the perivitelline space (PVS) between the oocyte and the zona pellucida (ZP); MII—absence of a GV within the oocyte and presence of a PB in the PVS between the oocyte and the ZP.

Experiments 1 and 2, oocytes were individually imaged using brightfield microscopy on the ECHO Revolve. The images were later scored by a single trained embryologist according to the Total Oocyte Score (TOS) grading system (Lazzaroni-Tealdi *et al.*, 2015). Oocytes were given a score of −1, 0, or 1 for each of the following criteria: morphology, cytoplasmic granularity, PVS, ZP size, PB size, and oocyte diameter. ZP and oocyte diameter were measured using ECHO Revolve Microscope software and image analysis software Fiji (2.9.0/1.53t). The sum of all categories gave each oocyte a TOS (ranging from −6 to +6) with higher scores indicating better morphological quality. Oocytes were individually placed in 0.2 ml tubes containing 5 µl Dulbecco’s phosphate-buffered saline (DPBS), flash frozen in liquid nitrogen, and stored at −80°C for future molecular analysis.

For Experiment 3, individual oocyte imaging and scoring was not performed in order to minimize handling and disruption of oocytes prior to fertilization and embryo culture.

### In vitro fertilization and embryo culture

For Experiment 3, COCs were cultured as outlined above for 28 h then denuded, assessed for MII formation, and imaged as a group. Individual oocytes were inseminated with sperm from one of three sperm donors via ICSI on Day 1 post-retrieval using sperm of suitable quality (Palermo *et al.*, 2014), then cultured in an Embryo Culture Medium (Global Total, Cooper Surgical, Bedminster, NJ) at 37 °C in a tri-gas incubator with CO_2_ adjusted so that the pH of the bicarbonate-buffered medium was 7.2–7.3, with the O_2_ level at 5%. At 16- to 18-h post-ICSI, fertilization was assessed and zygotes were imaged, and fertilized oocytes were cultured until Day 3. Cleaved embryos were imaged and underwent laser-assisted zona perforation and further cultured to develop until the blastocyst stage (de Boer *et al.*, 2004; McArthur *et al.*, 2005). Blastocysts were assessed on Day 5, 6 and/or 7, imaged, and scored according to the Gardner scale, and then underwent trophectoderm biopsy for preimplantation genetic testing for aneuploidy (PGT-A) if deemed to be of usable quality (i.e. greater than or equal to a 3CC rating) (Gardner *et al.*, 2000). Oocytes that failed to mature, failed to fertilize, failed to cleave, or failed to generate blastocysts of biopsy quality were flash frozen and sent for aneuploid analysis via PGT-A. No oocytes from this study were utilized or banked for transfer, implantation, or reproductive purposes.

Trophectoderm biopsies were transferred to 0.2 ml PCR tubes and sent to an external research laboratory (Juno Genetics, Basking Ridge, NJ) for comprehensive chromosomal analysis using a single nucleotide polymorphism-based next generation sequencing of all 46 chromosomes (PGT-A) (Handyside, 2013; Treff *et al.*, 2013).

Whole remaining blastocysts from the TE-biopsy PGT-A tested samples were harvested for epigenetic analysis through enzymatic methylation sequencing (EM-Seq).

### Blastocyst epigenetic analysis

Five euploid Day 5 or 6 blastocysts from the OSC-IVM condition and eight donated Day 5 of 6 euploid blastocysts from an COS reference control were flash frozen in 5 µl DPBS in 0.2 ml PCR tubes and transferred to an external research laboratory for methylation sequencing preparation (Azenta/Genewiz, NJ, USA). Blastocysts were individually harvested for high molecular weight genomic DNA (gDNA) extraction using the PicoPure DNA extraction kit (ThermoFisher) with an RNA carrier spike-in for yield improvement. Isolated gDNA was then prepared for epigenetic sequencing using the Enzymatic Methylation Sequencing Kit (EM-Seq) (NEB) (Vaisvila *et al.*, 2021). Individual embryos were sequenced at a depth of 100 million reads using a 2×150 bp Illumina kit on the Illumina NovaSeq instrument. Methylation analysis was performed as briefly described. Methylation alignments were constructed using a well-structured methylation analysis pipeline previously described (https://nf-co.re/methylseq/2.4.0) (Ewels *et al.*, 2020). Briefly, bismarck was utilized to map to the human reference genome (Hg37), selected to match to reference data (Okae *et al.*, 2014; Saenz-de-Juano *et al.*, 2019). A list of germline differentially methylated regions (gDMRs) was obtained from previous studies (Saenz-de-Juano *et al.*, 2019) and percent 5-methylcytosine (5mC) was calculated for each gDMR and plotted versus external literature controls. Additionally, whole genome 5mC levels were calculated and compared to the COS internal reference blastocysts as a model of standard fertility treatment embryos.

### Data analysis and statistics

Oocyte maturation outcome data were analyzed using Python statistical packages pandas (1.5.2), scipy (1.9.3), and statsmodels (0.13.5). Maturation percentages and embryo formation outcomes by donor group were analyzed by *t*-test as functions of the IVM environment (OSC-IVM or Media control). Two-tailed *t*-test statistics were computed comparing experimental versus control with Welch’s correction for unequal variance paired by donor. When comparing three independent groups one-way analysis of variance (ANOVA) was utilized. Embryo formation outcomes were analyzed by logistic regression, comparing outcomes per COC subjected to IVM (OSC-IVM vs Control), and significance was based on the OSC-IVM distribution compared to the Control mean. Bar graphs depict mean values and error bars represent SEM. Number of independent oocytes for the experiment, and paired versus unpaired *t*-test parameters are indicated above and in the figure legends.

### Prospective sample size estimation

Experiment 1 was a pilot analysis of the OSC-IVM and control culture conditions to determine the mean maturation rate and standard error. Based on these outcomes, we projected that 20 subjects and/or 80 oocytes per group would be a sufficient sample size for statistical measurement of the oocyte maturation rate with an 80% power. Based on the outcomes of Experiments 1 and 2, for Experiment 3, we estimated that 20 donors and/or a minimum of 65 oocytes in each group would allow for sufficient sample size for statistical measurement of euploid embryo formation with an 80% power.

## Results

### hiPSC-derived OSCs effectively promote human oocyte maturation in co-culture system

To obtain immature COCs, we utilized similar protocols to previous IVM studies, truncated IVF, or hCG primed-IVM: abbreviated gonadotropin stimulation (3–4 days) most often with an hCG trigger (Lin *et al.*, 2003; De Vos *et al.*, 2016; Sanchez *et al.*, 2019). This abbreviated stimulation program, particularly with higher doses of hCG (i.e. 10 000 IU), yielded a mixed cohort of COCs with different magnitudes of cumulus expansion. In Experiment 1, the rate of expanded COCs utilized in IVM was 14% ± 5.7% SEM in OSC-IVM, 13% ± 5.9% SEM in FOSC-IVM, and 20% ± 7.2% SEM in Media Control IVM, which did not significantly differ between groups (*P *=* *0.7156, ANOVA). In Experiment 2, the rate of expanded COCs utilized in IVM was 12.3% ± 3.9% SEM in OSC-IVM and 12.3% ± 4.9% SEM in the Commercial IVM control, which did not significantly differ between groups (*P *=* *0.99, paired *t*-test). In Experiment 3, for both OSC-IVM and Commercial IVM control the rate of expanded COCs utilized in IVM was 3.17% ± 3.17%, which did not significantly differ between groups (*P *=* *0.99, paired *t*-test).

Oocyte donor demographics and treatment regimens are shown in Table 2 for each experimental group. Overall, the results demonstrate that we were able to retrieve oocytes from donors, albeit at a lower yield than traditional ovarian stimulation cycles. Follicle measurements taken during stimulation in all subjects shows that extraction occurred in follicles less than 6 mm on an average for 33.03% ± 3.02% SEM, between 6 and 12 mm for 64.87% ± 3.16% SEM, and above 12 mm for 2.09% ± 0.63% SEM. Serum levels of E2, P4, and LH at OPU were similar in donors between groups within Experiment 1, but different across Experiments 1, 2, and 3. These hormonal values show evidence of early follicular growth with minimal instances of premature luteinization (Table 2).

**Table 2.** Donor demographic and stimulation characteristics.

	Experiment 1: OSC activity			Experiment 2: OSC clinical relevance	Experiment 3: OSC clinical relevance
	OSC-IVM group	FOSC-IVM group	Control group	IVM only	Embryo formation
Number of oocyte donors included	12	11	18	21	14
Oocyte donor age (Avg±SEM)	26 ± 0.94	28 ± 0.83	24 ± 1.1	29 ± 0.63	31.1 ± 0.69
Oocyte donor BMI (Avg±SEM)	22 ± 1.2	22 ± 0.51	22 ± 0.52	22 ± 0.58	25.3 ± 1.50
Total rFSH used for stimulation (IU)	325–600	325–600	325–600	600	5 days Clomid plus 150–300 IU or 600 IU
hCG trigger utilized (IU)	10 000	10 000	10 000	10 000	0 IU or 2500 IU
COCs obtained (Mean±SEM)	7.8 ± 0.98	8.5 ± 1.01	7.7 ± 1.03	7.0 ± 0.64	9.5 ± 1.40
AMH (ng/ml) (Mean±SEM)	5.1 ± 0.65	5.1 ± 0.81	4.2 ± 0.53	4.1 ± 0.42	6.4 ± 0.86
AFC (Mean±SEM)	17.7 ± 1.28	16.7 ± 0.98	15.1 ± 1.09	20.1 ± 0.12	27.9 ± 2.05
E2 at OPU (ng/ml) (Mean±SEM)	330 ± 52.4	308 ± 61.7	159 ± 52.5	474 ± 59.7	387 ± 135.4
P4 at OPU (ng/ml) (Mean±SEM)	0.69 ± 0.12	0.71 ± 0.10	0.98 ± 0.16	1.02 ± 0.12	1.1 ± 0.17
LH at OPU (ng/ml) (Mean±SEM)	4.35 ± 0.99	4.48 ± 0.90	5.27 ± 1.10	5.01 ± 0.90	8.75 ± 3.3

AFC, antral follicle count; AMH, anti-Mullerian hormone; BMI, body mass index; Clomid, clomiphene citrate; COCs, cumulus–oocyte complexes; E2, estradiol; FOSC, fetal-like ovarian somatic cells; hCG, human chorionic gonadotropin; IU, international units; IVM, *in vitro* maturation; LH, luteinizing hormone; OPU, oocyte retrieval; OSCs, ovarian support cells; P4, progesterone; rFSH, recombinant follicle stimulating hormone; SEM, standard error of the mean.

In Experiment 1, oocytes from each donor were allocated to two simultaneous culture conditions (control IVM and FOSC-IVM or OSC-IVM) when possible. For Experiments 2 and 3, the control and OSC-IVM arms contained paired sibling oocytes from each donor. A depiction of the three experimental designs is shown (Fig. 1A–C and Supplementary Fig. S1), including a representative image of the OSC co-culture (Fig. 1D).

We have previously demonstrated that hiPSC-derived OSCs are predominantly composed of granulosa-like cells and can be generated through overexpression of different TF combinations (Pierson Smela *et al.*, 2023). In response to hormonal stimulation treatment *in vitro*, the OSCs produce the extracellular matrix remodelers, growth factors, and steroids necessary for paracrine interaction with oocytes and cumulus cells (Pierson Smela *et al.*, 2023). To investigate whether hiPSC-derived OSCs are functionally capable of promoting human oocyte maturation *in vitro*, we established a co-culture system of these cells with freshly retrieved COCs and assessed oocyte maturation rates after 24–28 h (Experiment 1). We likewise tested a fetal-like version of OSCs (FOSC-IVM) to determine whether the adult OSC-IVM variant was specific in promoting maturation or whether other cell co-cultures could likewise promote maturation.

**Figure 2.**
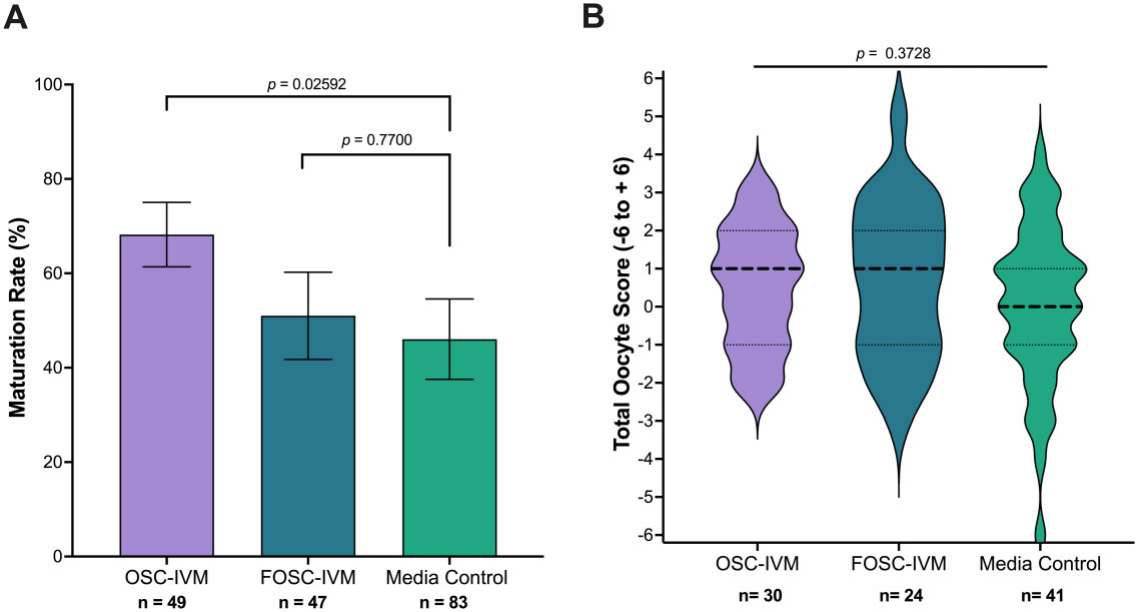
**OSCs improve human oocyte maturation rates compared to supplemented IVM medium lacking OSCs or containing other cell types.** (**A**) Maturation rate of oocytes after 24- to 28-h *in vitro* maturation (IVM) experiments in Experiment 1, including oocytes co-cultured with ovarian support cells (OSCs), fetal-like ovarian somatic cells (FOSCs) or in Media Control. n indicates the number of individual oocytes in each culture condition. Error bars indicate mean±SEM. *P*-value derived from unpaired *t-*test comparing OSC-IVM to control (Media Control) and FOSC-IVM to control (Media Control). (**B**) Total Oocyte Score (TOS) generated from imaging analysis of metaphase II (MII) oocytes after 24- to 28-h IVM experiments. n indicates the number of individual MII oocytes analyzed. Median (dashed line) and quartiles (dotted line) are indicated. A one way analysis of variance (ANOVA) indicated no significant difference between the means of the three groups. Due to low numbers of retrieved oocytes per donor, oocytes could not be consistently split between both conditions. Groups contain oocytes from predominantly non-overlapping donor cohorts thus pairwise comparisons are not utilized.

In this comparison, due to low numbers of retrieved oocytes per donor, we were unable to consistently split oocytes between all conditions simultaneously. Therefore, each group contains oocytes from predominantly non-overlapping donor groups and pairwise comparisons are not utilized. Strikingly, we observed a statistically significant improvement (∼1.5×) in maturation outcomes for oocytes that underwent IVM with OSC-IVM (Fig. 2A) compared to the Media Control. More specifically, the OSC-IVM group yielded a maturation rate of 68% ± 6.83% SEM versus 46% ± 8.51% SEM in the Media Control (Fig. 2A, *P *=* *0.02592, unpaired *t-*test). The FOSC-IVM did not yield significant improvement in maturation rate over the Media Control, yielding 51% ± 9.23% SEM (Fig. 2A, *P *=* *0.77 unpaired *t*-test). These results support the functional activity of specifically hiPSC-derived adult-like OSCs significantly improving oocyte maturation using this *in vitro* co-culture system. We next examined whether hiPSC-derived OSCs would also affect the outcome of oocyte morphological quality as assessed by the TOS. Interestingly, the assessment scores were not statistically significantly different for the three groups (Fig. 2B; ANOVA, *P *=* *0.3728), indicating that the mature MII oocytes were of equivalent morphological quality between the IVM conditions. Altogether, these data indicate that OSC co-culture improves human oocyte maturation without a detrimental effect on morphological quality and highlights the potential for the use of hiPSC-derived OSCs as a high performing system for cumulus enclosed oocyte IVM.

### Oocyte maturation rates in OSC-IVM outperforms commercially available IVM system

**Figure 3.**
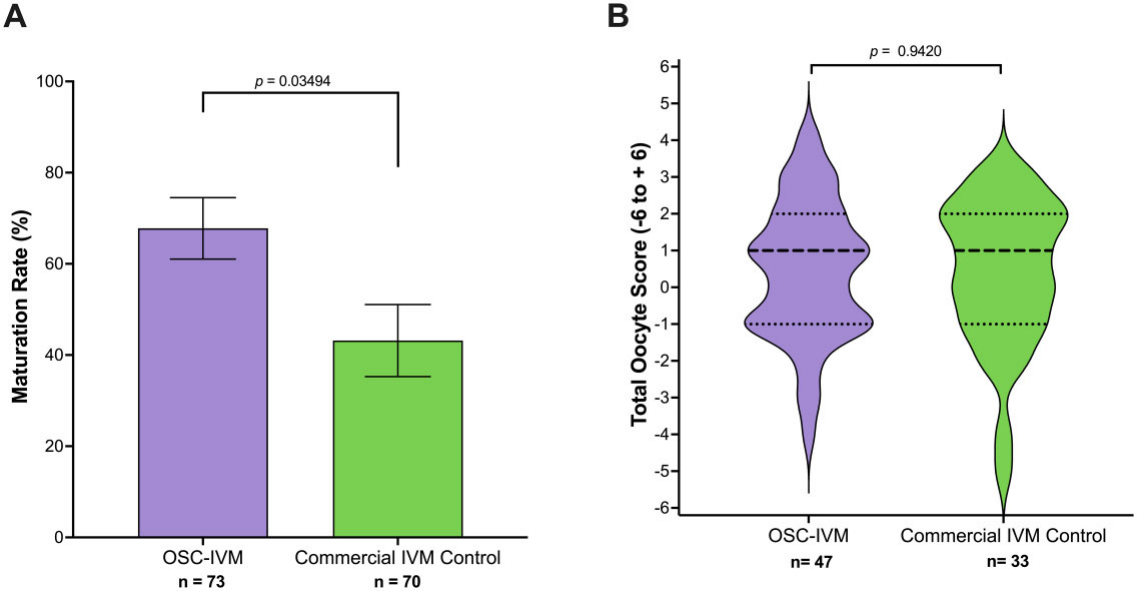
**OSC-IVM demonstrates improved oocyte maturation compared to a commercially available IVM system.** (**A**) Maturation rate of oocytes after 28-h *in vitro* maturation (IVM) experiments in Experiment 2, including oocyte co-culture with ovarian support cells (OSCs) or in the commercial IVM Control. n indicates the number of individual oocytes in each culture condition. Error bars indicate mean±SEM. *P*-value derived from paired *t*-test comparing Experimental OSC-IVM to the commercial IVM Control. (**B**) Total Oocyte Score (TOS) derived from imaging analysis of metaphase II (MII) oocytes after 28-h IVM experiments. n indicates the number of individual MII oocytes analyzed. Median (dashed line) and quartiles (dotted line) are indicated. An unpaired *t*-test indicated no significant (*P*=0.9420) difference between the means. Cumulus–oocyte complexes (COCs) from each donor were randomly and equitably distributed between control and intervention to allow for pairwise statistical comparison.

To further examine the potential for using OSC-IVM as a viable system to mature human oocytes in a more clinical setting, we compared our OSC co-culture system against a commercially available IVM standard, used according to the manufacturer’s instructions for use, with no modification (Medicult IVM, Cooper Surgical). We performed a sibling oocyte study comparing the MII formation rate and oocyte morphological quality after 28 h of IVM (Experiment 2). Notably, OSC-IVM yielded a statistically significant ∼1.6× higher average MII formation rate (68% ± 6.74% of mature oocytes in OSC-IVM versus 43% ± 7.90% in the control condition; Fig. 3A, *P *=* *0.0349, paired *t-*test). Similar to our prior observations, co-culture with hiPSC-derived OSCs did not affect oocyte morphological quality as measured by TOS, indicating equivalent oocyte morphological characteristics (Fig. 3B, *P* = 0.9420, unpaired *t-*test). These results show that OSC-IVM significantly outperformed the commercially available IVM culture medium in MII formation rate with no apparent detriment to oocyte morphological quality, pointing to a beneficial application of this product for human IVM.

### Cumulus enclosed immature oocytes from abbreviated gonadotropin stimulation matured by OSC-IVM are developmentally competent for embryo formation

**Figure 4.**
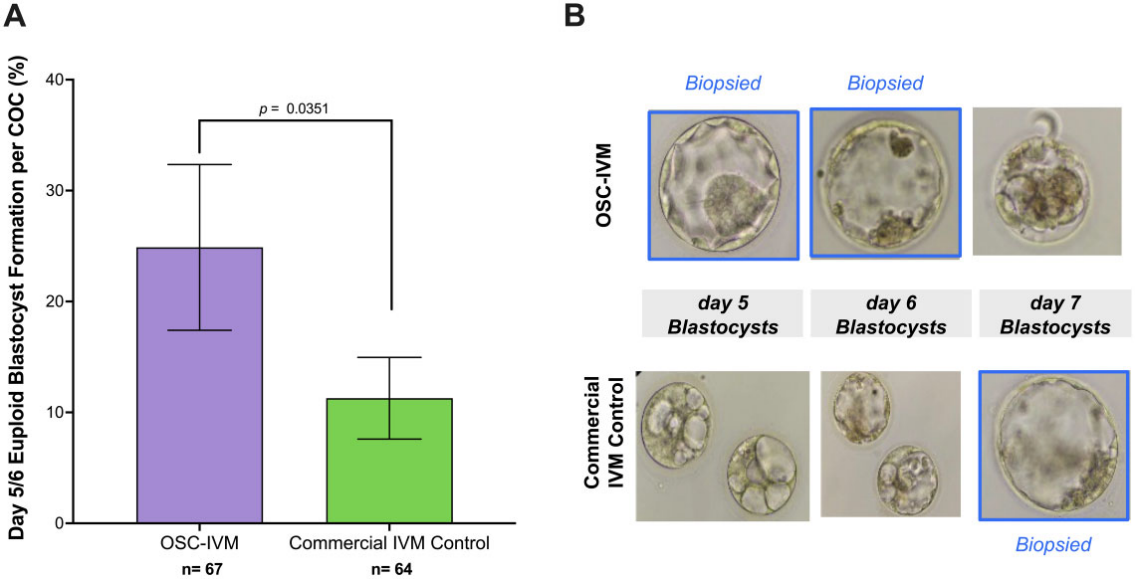
**OSC-IVM assisted oocytes are developmentally competent for healthy embryo formation.** (**A**) Euploid blastocyst (Day 5 or 6) formation rate per cumulus–oocyte complex (COC) in Experiment 3 after oocyte co-culture with ovarian support cells (OSCs) or in the commercial *in vitro* maturation (IVM) Control. n indicates the number of individual oocytes in each culture condition. Error bars indicate mean±SEM. *P*-value derived from logistic regression comparing Experimental OSC-IVM to the commercial IVM Control. (**B**) Representative images of embryo formation in OSC-IVM versus commercial IVM conditions at Days 5, 6, and 7 of blastocyst formation. Embryos that were of suitable vitrification quality are labeled as ‘biopsied’ and were utilized for trophectoderm biopsy and preimplantation genetic testing for aneuploidy (PGT-A).

We next investigated the developmental competency of oocytes treated in the OSC-IVM system by assessing euploid blastocyst formation after insemination, compared to the commercially available IVM control. Utilizing a limited cohort of 17 subjects who underwent abbreviated stimulation (Experiment 3, Table 1, and Table 2), we investigated whether OSC-IVM treated oocytes were capable of fertilization, cleavage, and formation of euploid blastocysts. Of the 17 subjects, three were excluded due to low oocyte retrieval yield (≤2 oocytes), which prevented pairwise comparison. As expected, OSC-IVM yielded a significantly higher ∼1.3× higher average MII formation rate with 56% ± 9.2% of mature oocytes compared to 43% ± 6.4% in the control condition (*P *=* *0.0476, logistic regression) (Table 3). Matured oocytes from both conditions were immediately utilized for ICSI and followed until embryo formation (blastocyst stage). Per MII oocyte, OSC-IVM oocytes displayed similar fertilization and cleavage rates (70% ± 10.2% and 70% ± 10.2%) compared to the control (81% ± 9.3% and 73% ± 11.3%). OSC-IVM MIIs trended towards improved blastocyst formation rates (64% ± 10.2%) compared to the control (50.7% ± 11.3%). OSC-IVM yielded a trend towards improvement in Day 5 or 6 blastocyst formation per COC (28% ± 7.6%) compared to the commercial IVM control (15% ± 4.4%) (Fig. 4, Table 3). Strikingly, OSC-IVM yielded a statistically significant improvement in Day 5 or 6 euploid blastocyst formation per COC (25% ± 7.5%) compared to the commercial IVM control (11% ± 3.8%) (Fig. 4, Table 3). These findings demonstrate that OSC-IVM generates healthy matured oocytes with high quality developmental competency. These results additionally demonstrate OSC-IVM is capable of producing healthy, euploid embryos from abbreviated stimulation cycles at a higher rate than the commercially available IVM condition, highlighting the clinical relevance of this novel system for IVM ART practice.

**Table 3.** OSC-IVM oocytes are developmentally competent for euploid embryo formation.

N* *=* *14 oocyte donors	OSC-IVM	Commercial IVM control	*P*-value (logistic regression)
**Number of COCs (n)**	67	64	NA
**Maturation (MII) rate (mean** **±** **SEM)**	56%±9.15%	43%±6.35%	0.0476
**Fertilization rate per COC (mean** **±** **SEM)**	39%±8.09%	35%±6.60%	0.3814
**Cleavage rate per COC (mean** **±** **SEM)**	39%±8.09%	31%±6.93%	0.287
**Blastocyst rate per COC (mean**±**SEM)**	34%±7.81%	22%±5.81%	0.0861
**Day 5 or 6 blastocyst rate per COC (mean**±**SEM)**	28%±7.58%	15%±4.37%	0.0945
**Euploid Day 5 or 6 blastocyst rate per COC (mean**±**SEM)**	25%±7.47%	11%±3.82%^a^	0.0349

a One embryo yielded no test result upon initial and repeat biopsy.

COCs, cumulus–oocyte complexes; hCG, human chorionic gonadotropin; IVM, *in vitro* maturation; MII, metaphase II; OSCs, ovarian support cells; SEM, standard error of the mean.

### Euploid blastocysts from OSC-IVM show normal epigenetic profile compared to COS and CAPA-IVM

**Figure 5.**
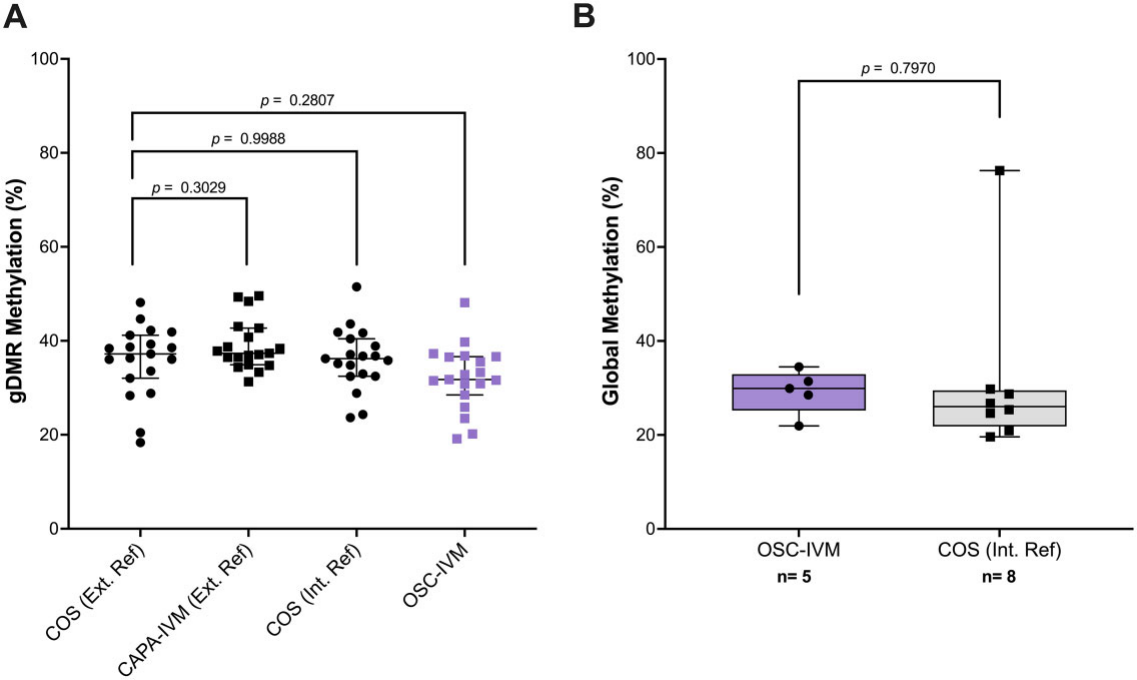
**OSC-IVM blastocysts display similar global and germline methylation profiles to COS blastocysts.** (**A**) 19 germline differentially methylated regions (gDMRs) were profiled for their methylation pattern in five ovarian support cell *in vitro* maturation (IVM) (OSC-IVM) Day 5 or 6 euploid blastocysts and eight donated Day 5 or 6 euploid blastocysts after conventional ovarian stimulation (COS). Additional control data were obtained from Saenz-de-Juano *et al.* (2019) for biphasic IVM (CAPA-IVM) and COS samples. Symbols represent gDMR percentages of individual embryos. Data are plotted as median with error bars representing 95% confidence interval (CI). Statistical significance testing was performed using one way analysis of variance (ANOVA) with multiple comparisons testing for *post hoc* analysis comparison to the external reference COS control. (**B**) Whole genome methylation analysis of 5-methylcytosine (5mC) levels for the five OSC-IVM blastocysts and eight donated COS blastocyst controls. Statistical significance testing was performed using two-way unpaired *t*-test comparison to the internal reference COS control.

Using five euploid Day 5 or 6 blastocysts generated from OSC-IVM and an additional eight donated controls obtained from COS and treatment, we assessed embryo epigenetic quality through EM-Seq. We further utilized external reference data generated on COS and other IVM approaches (CAPA-IVM) as further comparison controls (Saenz-de-Juano *et al.*, 2019). Our results showed that OSC-IVM generates embryos with no significant difference in 5mC methylation profile across 19 well-characterized gDMRs (Fig. 5A, ANOVA with multiple comparisons tests, *P *=* *0.2807). Furthermore, whole genome 5mC profiling showed that OSC-IVM embryos showed no significant difference compared to COS internal reference controls (Fig. 5B, unpaired *t*-test, *P *=* *0.7970). Together, these results indicate that OSC-IVM is not only capable of improving oocyte maturation and improving euploid Day 5 and 6 blastocyst formation, but it also generates embryos with global epigenetic profiles similar to embryos generated from conventional COS and other IVM treatments.

## Discussion

It is well established that immature oocytes obtained from human ovarian follicles can mature upon extraction and *in vitro* culture (Pincus and Enzmann, 1935; Edwards, 1965; Jie *et al.*, 2022). However, the rate and extent of oocyte maturation using these conditions are not optimized, resulting in highly unsynchronized nuclear and cytoplasmic maturation, and leaving IVM as an underperforming and underutilized technology. Current commercial IVM media have incrementally improved this process. With further optimization and development of additional culture tools, it is possible to improve the rate and extent of oocyte maturation, yielding high quality oocytes capable of healthy embryo formation. In this study, we investigate a novel IVM approach using human stem cell-derived OSCs as a co-culture system for IVM of human oocytes. We demonstrated that this OSC-IVM system outperforms media without OSCs and other cell lines as well as a commercially available IVM culture media for MII formation. This work is the first of its kind to explore the potential of stem cell-derived OSCs as a tool for IVM of human COCs and shows the value of this and other cell engineering approaches for application in fertility treatment. This study also demonstrated that the OSCs specifically, and not just cell co-culture in general, provided a benefit to the oocytes beyond that of the acellular supplements to the culture conditions (hormones, steroids, doxycycline). Therefore, the engineered granulosa-like cells are a beneficial addition to the *in vitro* culture environment for oocytes.

Notably, many previous IVM studies stopped at the embryo cleavage stage, whereas present ART practice increasingly relies heavily upon culture to the blastocyst stage and PGT-A as an embryo selection tool. Our study employs these more advanced selection tools. The OSC-IVM system results in MII oocytes with a high degree of developmental competence, as demonstrated by their ability to achieve fertilization, to become embryos capable of cell divisions, and to form blastocysts characterized with a normal euploid complement of chromosomes and normal epigenetic profile. In short, oocytes matured in the presence of OSCs are capable of becoming healthy euploid embryos that would likely be competent to implant and become live born babies. The ability to improve the formation of healthy, euploid blastocysts is further evidence of the ability of the OSC-IVM technique to promote cytoplasmic maturation as well as nuclear maturation of oocytes, a key requirement for clinical utility of IVM systems. Further studies will explore this hypothesis. The high euploidy rate is a key indication of the potential clinical utility of OSC-IVM compared to conventional IVM systems.

This method of IVM involving co-culture integrates readily with existing procedures employed in ART laboratories, requiring no special equipment and only minimal training for clinical embryologists. The OSC-IVM approach is generated from a single source of highly qualified, commercially available hiPSCs and does not require patient-specific generation. This approach would therefore be highly scalable, reproducible, and cost-effective as a novel IVM treatment for patients as opposed to individualized primary cell co-culture or COS treatment. Likewise, OSC-IVM performs effectively in hCG-triggered cycles with variable follicle size, which is an improvement over other IVM systems that are ill-suited for these more common stimulation styles (Sanchez *et al.*, 2019). Our results show that OSC-IVM can be utilized across a variety of stimulation regimens, follicle extraction methodologies, and without hCG triggers, making the system highly amenable to customized abbreviated stimulation programs across the spectrum from strict IVM to truncated IVF practice. Additionally, the paracrine interaction between the COCs and OSCs in this system, in which direct connections/gap junctions are not formed, affords a high degree of flexibility in co-culture format delivery, and allows for a broad range of potential culture configurations.

This study represents an important first step in establishing stem cell derived OSCs as a co-culture platform for IVM. This study builds on previous findings that demonstrate a benefit to using primary granulosa cell co-culture for IVM of oocytes (Torre *et al.*, 2006; Johnson *et al.*, 2008; Jahromi *et al.*, 2015; Zgórecka *et al.*, 2022). The use of OSCs derived from hiPSCs represents a powerful approach for providing curated granulosa-like cells with characteristics more similar to a follicular, rather than luteal, phase environment, as well as providing a highly controlled and robust manner for their consistent production and qualification for broad use in clinical settings. Indeed, recent advances in IVM practice such as CAPA-IVM, which seeks to mimic aspects of granulosa cell function in the IVM culture environment through supplementation of CNP and AREG show that the biological role of granulosa cells in maturing oocytes *in vitro* is paramount to improving oocyte maturation rate and oocyte quality (Sanchez *et al.*, 2019; Vuong *et al.*, 2020a; Lee *et al.*, 2021). Those findings are largely consistent with the findings of this study that show direct supplementation of granulosa-like cells indeed improves oocyte maturation and developmental competence. While a direct comparison of OSC-IVM and CAPA-IVM was not performed in this study, the rates of oocyte maturation were similar for both OSC-IVM and CAPA-IVM as well as for the common commercial IVM control used in both studies. OSC-IVM results in a significant improvement in Day 5 and 6 euploid blastocyst formation rate, which was not demonstrated in previous CAPA-IVM studies, which stopped at the cleavage stage of embryo development. Future studies are warranted to explore differences in maturation and embryo formation rates between OSC-IVM and other IVM approaches.

### Limitations and reasons for caution

In general, while this study establishes a novel co-culture system for improving IVM outcomes compared to currently available IVM methods, a number of limitations and areas of future research remain. The nature of this study as a basic research study limits the generalizability of the findings, and future randomized control clinical evaluations will be important for establishing the value of the approach. Given that COCs were utilized from hCG-triggered cycles, the underlying maturation state of the oocytes prior to IVM could not be empirically assessed while maintaining the integrity of the cumulus-oocyte connections. Further, while equitable distribution of COCs between conditions was performed, slight imbalances in the GV/MI/MII input distribution may represent a limitation of the study. This study utilized relatively small sample sizes overall and larger studies of the approach will assist in clarifying the broader efficacy and safety. Further, additional studies should be performed to more precisely determine the mechanism of the approach and to characterize the specific secreted factors and changes to the *in vitro* growth environment that are responsible for the improved IVM performance. While it is well established that granulosa cell co-culture improves oocyte maturation in COCs and denuded oocytes, it will still be beneficial to determine how these hiPSC-derived OSCs perform their function and to compare them to primary cell co-culture systems (Torre *et al.*, 2006; Johnson *et al.*, 2008; Jahromi *et al.*, 2015). Lastly, important validation of the approach’s clinical utility will be the ability to generate healthy live births, and further clinical evaluation is warranted to determine whether such an approach provides clinical value for patients over existing IVM and COS treatments.

## Supplementary Material

dead205_Supplementary_Figure_S1

dead205_Supplementary_Table_S1

## Data Availability

All data needed to evaluate the conclusions in the paper are present in the paper and supplementary tables and figures. Raw and processed sequencing data will be deposited to GEO upon publication. Anonymized raw data for all findings in the paper will be provided upon request.

## References

[dead205-B1] Akin N , Le AH, Ha UDT, Romero S, Sanchez F, Pham TD, Nguyen MHN, Anckaert E, Ho TM, Smitz J et al Positive effects of amphiregulin on human oocyte maturation and its molecular drivers in patients with polycystic ovary syndrome. Hum Reprod 2021;37:30–43. 34741172 10.1093/humrep/deab237

[dead205-B2] Braam SC , Consten D, Smeenk JMJ, Cohlen BJ, Curfs MHJM, Hamilton CJCM, Repping S, Mol BWJ, de Bruin JP. In vitro maturation of oocytes in women at risk of ovarian hyperstimulation syndrome—a prospective multicenter cohort study. Int J Fertil Steril 2019;13:38–44. 30644243 10.22074/ijfs.2019.5452 PMC6334011

[dead205-B3] Braam SC , de Bruin JP, Mol BWJ, van Wely M. The perspective of women with an increased risk of OHSS regarding the safety and burden of IVF: a discrete choice experiment. Hum Reprod Open 2020;2020:hoz034. 32123754 10.1093/hropen/hoz034 PMC7039283

[dead205-B4] de Boer KA , Catt JW, Jansen RPS, Leigh D, McArthur S. Moving to blastocyst biopsy for preimplantation genetic diagnosis and single embryo transfer at Sydney IVF. Fertil Steril 2004;82:295–298. 15302271 10.1016/j.fertnstert.2003.11.064

[dead205-B5] De Vos M , Grynberg M, Ho TM, Yuan Y, Albertini DF, Gilchrist RB. Perspectives on the development and future of oocyte IVM in clinical practice. J Assist Reprod Genet 2021;38:1265–1280. 34218388 10.1007/s10815-021-02263-5 PMC8266966

[dead205-B6] De Vos M , Ortega-Hrepich C, Albuz FK, Guzman L, Polyzos NP, Smitz J, Devroey P. Clinical outcome of non-hCG-primed oocyte in vitro maturation treatment in patients with polycystic ovaries and polycystic ovary syndrome. Fertil Steril 2011;96:860–864. 21868003 10.1016/j.fertnstert.2011.07.1108

[dead205-B7] De Vos M , Smitz J, Thompson JG, Gilchrist RB. The definition of IVM is clear-variations need defining. Hum Reprod 2016;31:2411–2415. 27664204 10.1093/humrep/dew208

[dead205-B8] De Vos M , Smitz J, Woodruff TK. Fertility preservation in women with cancer. Lancet 2014;384:1302–1310. 25283571 10.1016/S0140-6736(14)60834-5 PMC4270060

[dead205-B9] Edwards RG. Maturation in vitro of mouse, sheep, cow, pig, rhesus monkey and human ovarian oocytes. Nature 1965;208:349–351. 4957259 10.1038/208349a0

[dead205-B10] El Tokhy O , Kopeika J, El-Toukhy T. An update on the prevention of ovarian hyperstimulation syndrome. Womens Health (Lond) 2016;12:496–503. 27543490 10.1177/1745505716664743 PMC5373266

[dead205-B11] Erickson GF , Shimasaki S. The physiology of folliculogenesis: the role of novel growth factors. Fertil Steril 2001;76:943–949. 11704115 10.1016/s0015-0282(01)02859-x

[dead205-B12] Ethics Committee of the American Society for Reproductive Medicine. Electronic address: asrm@asrm.org. Disparities in access to effective treatment for infertility in the United States: an Ethics Committee opinion. Fertil Steril 2021;116:54–63. 34148590 10.1016/j.fertnstert.2021.02.019

[dead205-B13] Ewels PA , Peltzer A, Fillinger S, Patel H, Alneberg J, Wilm A, Garcia MU, Di Tommaso P, Nahnsen S. The NF-core framework for community-curated bioinformatics pipelines. Nat Biotechnol 2020;38:276–278. 32055031 10.1038/s41587-020-0439-x

[dead205-B14] Fadini R , Dal Canto MB, Mignini Renzini M, Brambillasca F, Comi R, Fumagalli D, Lain M, Merola M, Milani R, De Ponti E. Effect of different gonadotrophin priming on IVM of oocytes from women with normal ovaries: a prospective randomized study. Reprod Biomed Online 2009;19:343–351. 19778479 10.1016/s1472-6483(10)60168-x

[dead205-B15] Fauser BC. Towards the global coverage of a unified registry of IVF outcomes. Reprod Biomed Online 2019;38:133–137. 30593441 10.1016/j.rbmo.2018.12.001

[dead205-B16] Fontana J , Martínková S, Petr J, Žalmanová T, Trnka J. Metabolic cooperation in the ovarian follicle. Physiol Res 2020;69:33–48. 31854191 10.33549/physiolres.934233 PMC8565957

[dead205-B17] Gardner DK , Lane M, Stevens J, Schlenker T, Schoolcraft WB. Blastocyst score affects implantation and pregnancy outcome: towards a single blastocyst transfer. Fertil Steril 2000;73:1155–1158. 10856474 10.1016/s0015-0282(00)00518-5

[dead205-B18] Gaskins AJ , Zhang Y, Chang J, Kissin DM. Predicted probabilities of live birth following assisted reproductive technology using United States national surveillance data from 2016 to 2018. Am J Obstet Gynecol 2023;228:557.e1. 10.1016/j.ajog.2023.01.014 PMC11057011 36702210

[dead205-B19] Grynberg M , Sermondade N, Sellami I, Benoit A, Mayeur A, Sonigo C. In vitro maturation of oocytes for fertility preservation: a comprehensive review. F&S Rev 2022;3:211–226.

[dead205-B20] Guzman L , Ortega-Hrepich C, Albuz FK, Verheyen G, Devroey P, Smitz J, De Vos M. Developmental capacity of in vitro-matured human oocytes retrieved from polycystic ovary syndrome ovaries containing no follicles larger than 6 mm. Fertil Steril 2012;98:503–507.e1–e2. 22365339 10.1016/j.fertnstert.2012.01.114

[dead205-B21] Handyside AH. 24-chromosome copy number analysis: a comparison of available technologies. Fertil Steril 2013;100:595–602. 23993662 10.1016/j.fertnstert.2013.07.1965

[dead205-B22] Hsieh M , Zamah AM, Conti M. Epidermal growth factor-like growth factors in the follicular fluid: role in oocyte development and maturation. Semin Reprod Med 2009;27:52–61. 19197805 10.1055/s-0028-1108010 PMC4624340

[dead205-B23] Jahromi BN , Mosallanezhad Z, Matloob N, Davari M, Ghobadifar MA. The potential role of granulosa cells in the maturation rate of immature human oocytes and embryo development: a co-culture study. Clin Exp Reprod Med 2015;42:111–117. 26473111 10.5653/cerm.2015.42.3.111 PMC4604294

[dead205-B24] Jie H , Zhao M, Alqawasmeh OAM, Chan CPS, Lee TL, Li T, Chan DYL. In vitro rescue immature oocytes—a literature review. Hum Fertil (Camb) 2022;25:640–650. 33508986 10.1080/14647273.2021.1876932

[dead205-B25] Johnson JE , Higdon HL III, Boone WR. Effect of human granulosa cell co-culture using standard culture media on the maturation and fertilization potential of immature human oocytes. Fertil Steril 2008;90:1674–1679. 17953965 10.1016/j.fertnstert.2007.06.017

[dead205-B26] Lazzaroni-Tealdi E , Barad DH, Albertini DF, Yu Y, Kushnir VA, Russell H, Wu Y-G, Gleicher N. Oocyte scoring enhances embryo-scoring in predicting pregnancy chances with IVF where it counts most. PLoS One 2015;10:e0143632. 26630267 10.1371/journal.pone.0143632 PMC4668065

[dead205-B27] Lee AWT , Ng JKW, Liao J, Luk AC, Suen AHC, Chan TTH, Cheung MY, Chu HT, Tang NLS, Zhao MP et al Single-cell RNA sequencing identifies molecular targets associated with poor in vitro maturation performance of oocytes collected from ovarian stimulation. Hum Reprod 2021;36:1907–1921. 34052851 10.1093/humrep/deab100

[dead205-B48] Leung PCK, Adashi EY. *The ovary*. San Diego: Academic Press, an imprint of Elsevier, 2019.

[dead205-B28] Lin Y-H , Hwang J-L, Huang L-W, Mu S-C, Seow K-M, Chung J, Hsieh B-C, Huang S-C, Chen C-Y, Chen P-H. Combination of FSH priming and hCG priming for in-vitro maturation of human oocytes. Hum Reprod 2003;18:1632–1636. 12871873 10.1093/humrep/deg335

[dead205-B29] Liu W , Xin Q, Wang X, Wang S, Wang H, Zhang W, Yang Y, Zhang Y, Zhang Z, Wang C et al Estrogen receptors in granulosa cells govern meiotic resumption of pre-ovulatory oocytes in mammals. Cell Death Dis 2017;8:e2662. 28277543 10.1038/cddis.2017.82 PMC5386574

[dead205-B30] Ma L , Cai L, Hu M, Wang J, Xie J, Xing Y, Shen J, Cui Y, Liu XJ, Liu J. Coenzyme Q10 supplementation of human oocyte in vitro maturation reduces postmeiotic aneuploidies. Fertil Steril 2020;114:331–337. 32646587 10.1016/j.fertnstert.2020.04.002

[dead205-B31] McArthur SJ , Leigh D, Marshall JT, de Boer KA, Jansen RPS. Pregnancies and live births after trophectoderm biopsy and preimplantation genetic testing of human blastocysts. Fertil Steril 2005;84:1628–1636. 16359956 10.1016/j.fertnstert.2005.05.063

[dead205-B32] Mohsenzadeh M , Khalili MA, Anbari F, Vatanparast M. High efficiency of homemade culture medium supplemented with GDF9-β in human oocytes for rescue in vitro maturation. Clin Exp Reprod Med 2022;49:149–158. 35698778 10.5653/cerm.2021.05050 PMC9184876

[dead205-B33] Moschini RM , Chuang L, Poleshchuk F, Slifkin RE, Copperman AB, Barritt J. Commercially available enhanced in vitro maturation medium does not improve maturation of germinal vesicle and metaphase I oocytes in standard in vitro fertilization cases. Fertil Steril 2011;95:2645–2647. 21561612 10.1016/j.fertnstert.2011.03.094

[dead205-B34] Nilsson E , Skinner MK. Cellular interactions that control primordial follicle development and folliculogenesis. J Soc Gynecol Investig 2001;8:S17–S20. 10.1016/s1071-5576(00)00099-x 11223364

[dead205-B35] Osterman MJK, Hamilton BE, Martin JA, Driscoll AK, Valenzuela CP. *Births: Final data for 2021*. National Vital Statistics Reports. Hyattsville, MD: National Center for Health Statistics 2023;72. 36723449

[dead205-B36] Okae H , Chiba H, Hiura H, Hamada H, Sato A, Utsunomiya T, Kikuchi H, Yoshida H, Tanaka A, Suyama M et al Genome-wide analysis of DNA methylation dynamics during early human development. PLoS Genet 2014;10:e1004868. 25501653 10.1371/journal.pgen.1004868 PMC4263407

[dead205-B37] Palermo GD , Neri QV, Schlegel PN, Rosenwaks Z. Intracytoplasmic sperm injection (ICSI) in extreme cases of male infertility. PLoS One 2014;9:e113671. 25437298 10.1371/journal.pone.0113671 PMC4249967

[dead205-B38] Park J-Y , Su Y-Q, Ariga M, Law E, Jin S-LC, Conti M. EGF-like growth factors as mediators of LH action in the ovulatory follicle. Science 2004;303:682–684. 14726596 10.1126/science.1092463

[dead205-B39] Pierson Smela MD , Kramme CC, Fortuna PRJ, Adams JL, Su R, Dong E, Kobayashi M, Brixi G, Kavirayuni VS, Tysinger E et al Directed differentiation of human iPSCs to functional ovarian granulosa-like cells via transcription factor overexpression. Elife 2023;12:e83291. 36803359 10.7554/eLife.83291 PMC9943069

[dead205-B40] Pincus G , Enzmann EV. The comparative behavior of mammalian eggs in vivo and in vitro: I. The activation of ovarian eggs. J Exp Med 1935;62:665–675. 19870440 10.1084/jem.62.5.665 PMC2133299

[dead205-B41] Pongsuthirak P , Songveeratham S, Vutyavanich T. Comparison of blastocyst and Sage media for in vitro maturation of human immature oocytes. Reprod Sci 2015;22:343–346. 25015901 10.1177/1933719114542027 PMC4352143

[dead205-B42] Saenz-de-Juano MD , Ivanova E, Romero S, Lolicato F, Sánchez F, Van Ranst H, Krueger F, Segonds-Pichon A, De Vos M, Andrews S et al DNA methylation and mRNA expression of imprinted genes in blastocysts derived from an improved in vitro maturation method for oocytes from small antral follicles in polycystic ovary syndrome patients. Hum Reprod 2019;34:1640–1649. 31398248 10.1093/humrep/dez121

[dead205-B43] Sanchez F , Le AH, Ho VNA, Romero S, Van Ranst H, De Vos M, Gilchrist RB, Ho TM, Vuong LN, Smitz J. Biphasic in vitro maturation (CAPA-IVM) specifically improves the developmental capacity of oocytes from small antral follicles. J Assist Reprod Genet 2019;36:2135–2144. 31399916 10.1007/s10815-019-01551-5 PMC6823411

[dead205-B44] Sánchez F , Lolicato F, Romero S, De Vos M, Van Ranst H, Verheyen G, Anckaert E, Smitz JEJ. An improved IVM method for cumulus-oocyte complexes from small follicles in polycystic ovary syndrome patients enhances oocyte competence and embryo yield. Hum Reprod 2017;32:2056–2068. 28938744 10.1093/humrep/dex262

[dead205-B45] Shu-Chi M , Jiann-Loung H, Yu-Hung L, Tseng-Chen S, Ming-I L, Tsu-Fuh Y. Growth and development of children conceived by in-vitro maturation of human oocytes. Early Hum Dev 2006;82:677–682. 16690233 10.1016/j.earlhumdev.2006.01.012

[dead205-B46] Son W-Y , Tan SL. Laboratory and embryological aspects of hCG-primed in vitro maturation cycles for patients with polycystic ovaries. Hum Reprod Update 2010;16:675–689. 20504873 10.1093/humupd/dmq014

[dead205-B47] Strączyńska P , Papis K, Morawiec E, Czerwiński M, Gajewski Z, Olejek A, Bednarska-Czerwińska A. Signaling mechanisms and their regulation during in vivo or in vitro maturation of mammalian oocytes. Reprod Biol Endocrinol 2022;20:37. 35209923 10.1186/s12958-022-00906-5 PMC8867761

[dead205-B49] Torre ML , Munari E, Albani E, Levi-Setti PE, Villani S, Faustini M, Conte U, Vigo D. In vitro maturation of human oocytes in a follicle-mimicking three-dimensional coculture. Fertil Steril 2006;86:572–576. 16815387 10.1016/j.fertnstert.2006.02.090

[dead205-B50] Treff NR , Forman EJ, Scott RT Jr. Next-generation sequencing for preimplantation genetic diagnosis [review of next-generation sequencing for preimplantation genetic diagnosis]. Fertil Steril 2013;99:e17–e18. 23481279 10.1016/j.fertnstert.2013.02.034

[dead205-B51] Vaisvila R , Ponnaluri VKC, Sun Z, Langhorst BW, Saleh L, Guan S, Dai N, Campbell MA, Sexton BS, Marks K et al Enzymatic methyl sequencing detects DNA methylation at single-base resolution from picograms of DNA. Genome Res 2021;31:1280–1289. 34140313 10.1101/gr.266551.120 PMC8256858

[dead205-B52] Vuong LN , Ho VNA, Ho TM, Dang VQ, Phung TH, Giang NH, Le AH, Pham TD, Wang R, Smitz J et al In-vitro maturation of oocytes versus conventional IVF in women with infertility and a high antral follicle count: a randomized non-inferiority controlled trial. Hum Reprod2020a;35:2537–2547. 32974672 10.1093/humrep/deaa240

[dead205-B53] Vuong LN , Le AH, Ho VNA, Pham TD, Sanchez F, Romero S, De Vos M, Ho TM, Gilchrist RB, Smitz J. Live births after oocyte in vitro maturation with a prematuration step in women with polycystic ovary syndrome. J Assist Reprod Genet2020b;37:347–357. 31902102 10.1007/s10815-019-01677-6 PMC7056678

[dead205-B54] Walls ML , Hunter T, Ryan JP, Keelan JA, Nathan E, Hart RJ. In vitro maturation as an alternative to standard in vitro fertilization for patients diagnosed with polycystic ovaries: a comparative analysis of fresh, frozen and cumulative cycle outcomes. Hum Reprod 2015;30:88–96. 25355587 10.1093/humrep/deu248

[dead205-B55] Zgórecka W , Jeseta M, Prochazka R, Amorim CA, Krajnik K, Mozdziak P, Pieńskowski W, Skowroński MT, Kranc W. Approaches for in vitro culture of granulosa cells and ovarian follicles. Med J Cell Biol 2022;10:34–42.

[dead205-B56] Zhang H , Lu S, Xu R, Tang Y, Liu J, Li C, Wei J, Yao R, Zhao X, Wei Q et al Mechanisms of estradiol-induced EGF-like factor expression and oocyte maturation via G protein-coupled estrogen receptor. Endocrinology 2020;161:bqaa190. 33068422 10.1210/endocr/bqaa190

[dead205-B57] Zhang JJ , Merhi Z, Yang M, Bodri D, Chavez-Badiola A, Repping S, van Wely M. Minimal stimulation IVF vs conventional IVF: a randomized controlled trial. Am J Obstet Gynecol 2016;214:96.e1–e8. 10.1016/j.ajog.2015.08.009 26259908

