# Supplementary material for: Human-induced pluripotent stem cell-derived ovarian support cell co-culture improves oocyte maturation in vitro after abbreviated gonadotropin stimulation
Source: Hum Reprod. 2023 Oct 10;38(12):2456–69. doi: 10.1093/humrep/dead205 (PMC10694404; doi:10.1093/humrep/dead205)
Supplement: dead205_Supplementary_Figure_S1 [file dead205_supplementary_figure_s1.pdf]

Human Reproduction, 2023, 1–1
https://doi.org/10.1093/humrep/dead205
Supplementary Data

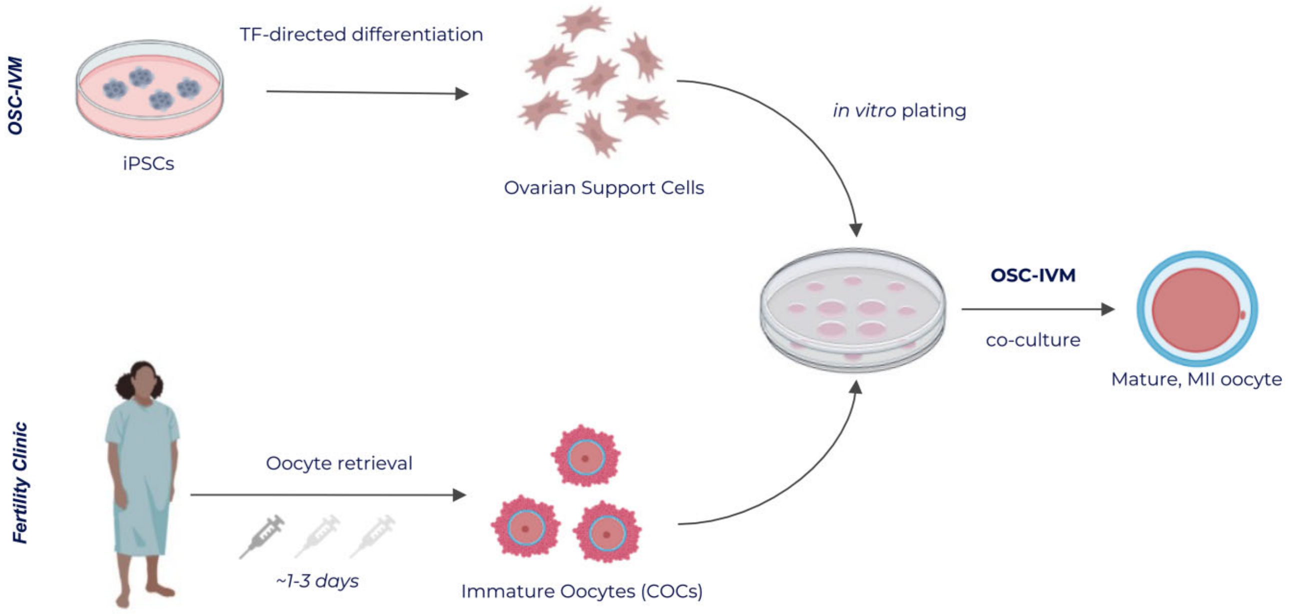

Supplementary Figure S1. Ovarian support cell (OSC) co-culture system. Schematic of the experimental co-culture *in vitro* maturation (IVM) approach. Human-induced pluripotent stem cells (hiPSCs) were differentiated using inducible transcription factor overexpression to form ovarian support cells (OSCs). Immature human cumulus–oocyte complexes (COCs) were obtained from donors in the clinic after undergoing abbreviated gonadotropin stimulation. In the lab, embryology dishes were prepared including OSCs seeding as required, and COCs were introduced for IVM co-culture. Oocyte maturation and morphological quality were assessed after 24- to 28-h IVM co-culture, and samples were either banked for analysis or utilized for embryo formation.
