# Supplementary material for: Human-induced pluripotent stem cell-derived ovarian support cell co-culture improves oocyte maturation in vitro after abbreviated gonadotropin stimulation
Source: Hum Reprod. 2023 Oct 10;38(12):2456–69. doi: 10.1093/humrep/dead205 (PMC10694404; doi:10.1093/humrep/dead205)
Supplement: dead205_Supplementary_Table_S1 [file dead205_supplementary_table_s1.pdf]

OXFORD
human reproduction

Human Reproduction, 2023, 1–1
https://doi.org/10.1093/humrep/dead205
Supplementary Data

Supplementary Table S1. Included donor characteristics and oocyte retrieval outcomes.

Oocyte donor clinic	Study ID	Donor age	Donor BMI	Clomid (mg)	rFSH (IU)	FSH regimen	hCG (IU)	Oocytes retrieved	Experiment
Pranor	P01	25	20.96	0	450	150-150-150	10 000	7	Experiment 1
Pranor	P02	25	18.03	0	450	150-150-150	10 000	6	Experiment 1
Pranor	P03	19	21.36	0	450	150-150-150	10 000	3	Experiment 1
Pranor	P04	24	23.23	0	375	125-125-125	10 000	3	Experiment 1
Pranor	P05	21	25.30	0	325	125-125-75	10 000	9	Experiment 1
Pranor	P06	23	24.46	0	325	125-125-75	10 000	11	Experiment 1
Ruber	R01	25	24.00	0	600	200-200-200	10 000	3	Experiment 1
Ruber	R02	20	22.00	0	600	200-200-200	10 000	10	Experiment 1
Ruber	R03	21	19.00	0	600	200-200-200	10 000	7	Experiment 1
Ruber	R04	37	19.00	0	600	200-200-200	10 000	12	Experiment 1
Ruber	R05	26	21.00	0	600	200-200-200	10 000	21	Experiment 1
Ruber	R06	23	20.00	0	450	150-150-150	10 000	8	Experiment 1
Ruber	R07	19	19.00	0	450	150-150-150	10 000	3	Experiment 1
Ruber	R08	31	Not determined	0	400	125-125-75-75	10 000	10	Experiment 1
Ruber	R09	20	22.00	0	400	125-125-75-75	10 000	7	Experiment 1
Ruber	R10	21	Not determined	0	400	125-125-75-75	10 000	7	Experiment 1
Ruber	R11	26	21.00	0	400	125-125-75-75	10 000	4	Experiment 1
Ruber	R12	31	24.00	0	600	200-200-200	10 000	7	Experiment 1
Ruber	R13	25	28.00	0	600	200-200-200	10 000	3	Experiment 1
Ruber	R16	31	20.00	0	600	200-200-200	10 000	14	Experiment 1
Ruber	R17	30	22.00	0	600	200-200-200	10 000	11	Experiment 1
Ruber	R18	31	20.55	0	600	300-150-150	10 000	6	Experiment 1
Ruber	R19	27	21.23	0	600	300-150-150	10 000	10	Experiment 1
Ruber	R22	25	23.44	0	600	300-150-150	10 000	7	Experiment 1
Ruber	R23	28	27.44	0	600	200-200-200	10 000	5	Experiment 1
Ruber	R24	25	Not determined	0	600	300-150-150	10 000	12	Experiment 1
Ruber	R25	29	22.66	0	600	300-150-150	10 000	11	Experiment 1
Ruber	R38	25	22.76	0	600	200-200-200	10 000	10	Experiment 2
Ruber	R39	27	22.98	0	600	200-200-200	10 000	7	Experiment 2
Ruber	R40	30	22.77	0	600	200-200-200	10 000	8	Experiment 2
Ruber	R41	28	24.38	0	600	200-200-200	10 000	4	Experiment 2
Ruber	R42	33	24.24	0	600	200-200-200	10 000	11	Experiment 2
Ruber	R43	34	26.23	0	600	200-200-200	10 000	7	Experiment 2
Ruber	R44	27	20.6	0	600	200-200-200	10 000	12	Experiment 2
Ruber	R45	28	17.04	0	600	200-200-200	10 000	11	Experiment 2
Ruber	R46	34	19.05	0	600	200-200-200	10 000	8	Experiment 2
Ruber	R47	29	18.36	0	600	200-200-200	10 000	2	Experiment 2
Ruber	R49	33	19.72	0	600	200-200-200	10 000	9	Experiment 2
Ruber	R50	28	24.84	0	600	200-200-200	10 000	2	Experiment 2
Ruber	R51	26	23.53	0	600	200-200-200	10 000	8	Experiment 2
Ruber	R52	30	23.03	0	600	200-200-200	10 000	8	Experiment 2
Ruber	R53	25	19.03	0	600	200-200-200	10 000	7	Experiment 2
Ruber	R54	31	24.22	0	600	200-200-200	10 000	4	Experiment 2
Ruber	R55	29	20.31	0	600	200-200-200	10 000	6	Experiment 2
Ruber	R56	27	21.3	0	600	200-200-200	10 000	3	Experiment 2
Ruber	R57	32	25.97	0	600	200-200-200	10 000	4	Experiment 2
Ruber	R58	32	25.34	0	600	200-200-200	10 000	5	Experiment 2
Ruber	R59	27	21.8	0	600	200-200-200	10 000	8	Experiment 2
Spring	SP02	26	22.70	500	150	150	2500	7	Experiment 3
Spring	SP03	33	23.40	500	300	150-150	2500	11	Experiment 3
Spring	SP04	33	19.10	500	300	150-150	2500	5	Experiment 3
Spring	SP05	30	21.60	500	300	150-150	0	11	Experiment 3
Spring	SP06	32	26.6	500	300	150-150	0	3	Experiment 3
Spring	SP09	29	24.10	500	300	150-150	0	4	Experiment 3
Spring	SP10	30	38.70	500	300	150-150	2500	8	Experiment 3
Spring	SP11	34	23.4	500	300	150-150	2500	9	Experiment 3
Spring	SP19	33	22.4	500	300	150-150	2500	13	Experiment 3
Spring	SP20	27	35.8	500	300	150-150	2500	9	Experiment 3
Spring	SP21	31	Not determined	500	300	150-150	2500	9	Experiment 3
Spring	SP22	34	23.03	500	300	150-150	2500	10	Experiment 3
Extend	EF04	30	23.91	0	600	200-200-200	2500	20	Experiment 3
Extend	EF03	34	24.03	0	600	200-200-200	2500	14	Experiment 3
